# In Vitro and In Vivo Validation of GATA-3 Suppression for Induction of Adipogenesis and Improving Insulin Sensitivity

**DOI:** 10.3390/ijms231911142

**Published:** 2022-09-22

**Authors:** Hend Al-Jaber, Nura A. Mohamed, Vijay K. Govindharajan, Samir Taha, Jomon John, Sharique Halim, Maha Alser, Shamma Al-Muraikhy, Najeha Rizwana Anwardeen, Abdelali Agouni, Abdelbary Elhissi, Hamda A. Al-Naemi, Layla Al-Mansoori, Mohamed A. Elrayess

**Affiliations:** 1 Biomedical Research Center, Qatar University, Doha P.O. Box 2713, Qatar; 2 Laboratory Research Animal Center, Qatar University, Doha P.O. Box 2713, Qatar; 3 Oral and Maxillofacial Surgery, Al-Wakra Hospital, Hamad Medical Corporation, Doha P.O. Box 3050, Qatar; 4 Department of Pharmaceutical Sciences, College of Pharmacy, QU Health, Qatar University, Doha P.O. Box 2713, Qatar; 5 Department of Biological and Environmental Sciences, College of Arts and Sciences, Qatar University, Doha P.O. Box 2713, Qatar; 6 College of Pharmacy, QU Health, Qatar University, Doha P.O. Box 2713, Qatar

**Keywords:** adipogenesis, insulin resistance, insulin sensitivity, subcutaneous fat, omental fat, type II diabetes mellitus

## Abstract

Impaired adipogenesis is associated with the development of insulin resistance and an increased risk of type 2 diabetes (T2D). GATA Binding Protein 3 (GATA3) is implicated in impaired adipogenesis and the onset of insulin resistance. Therefore, we hypothesize that inhibition of GATA3 could promote adipogenesis, restore healthy fat distribution, and enhance insulin signaling. Primary human preadipocytes were treated with GATA3 inhibitor (DNAzyme hgd40). Cell proliferation, adipogenic capacity, gene expression, and insulin signaling were measured following well-established protocols. BALB/c mice were treated with DNAzyme hgd40 over a period of 2 weeks. Liposomes loaded with DNAzyme hgd40, pioglitazone (positive), or vehicle (negative) controls were administered subcutaneously every 2 days at the right thigh. At the end of the study, adipose tissues were collected and weighed from the site of injection, the opposite side, and the omental depot. Antioxidant enzyme (superoxide dismutase and catalase) activities were assessed in animals’ sera, and gene expression was measured using well-established protocols. In vitro GATA3 inhibition induced the adipogenesis of primary human preadipocytes and enhanced insulin signaling through the reduced expression of p70S6K. In vivo GATA3 inhibition promoted adipogenesis at the site of injection and reduced MCP-1 expression. GATA3 inhibition also reduced omental tissue size and PPARγ expression. These findings suggest that modulating GATA3 expression offers a potential therapeutic benefit by correcting impaired adipogenesis, promoting healthy fat distribution, improving insulin sensitivity, and potentially lowering the risk of T2D.

## 1. Introduction

Obesity is at epidemic proportions with a steadily increasing prevalence that is expected to reach 300 million patients by 2025 [1]. In addition, obesity is a significant risk factor and a prominent contributor in the development of many pathological conditions, including cancer, cardiovascular diseases, diabetes (particularly type 2 (T2D)), liver and kidney diseases, and depression [2]. Impaired adipogenesis was linked to adipose tissue dysfunction and underlines the development of insulin resistance [3], and therefore T2D. During this process, subcutaneous adipose tissues (SAT) often have limited expandability, creating inappropriate adipocyte expansion, hypertrophic adipocyte features, the recruitment of inflammatory cells, and insulin-resistant phenotypes [4,5]. Numerous factors and pathways, including transcription factors, epigenetic regulators, signaling pathways, and inflammatory pathways, are involved in the processes of preadipocyte commitment and differentiation [6]. Hence, abnormalities in these pathways can result in the development of adipocyte dysfunction and insulin resistance, leading to related comorbidities, including T2D. 

Although the association between insulin resistance and obesity is well established [7,8], the link between impaired adipogenesis and insulin resistance remains unknown in non-obese individuals [9]. The dysfunction of SAT that includes adipocyte hypertrophy and the impairment of adipogenesis may play a significant role in the development of insulin resistance in non-obese individuals as it leads to fat deposition in the liver, skeletal muscle, and other fat depots [3,10,11]. Other studies have indicated that fat mass and distribution play a critical role in insulin resistance in non-obese individuals. In these studies, the large abdominal fat surface area was associated with an increased risk of insulin resistance, while the small size adipocytes was linked to the body’s insulin sensitivity [12], although the omental fat remains the only depot that correlates significantly with the metabolic syndrome [13]. The expression of high levels of cytokines by the hypertrophied adipocytes, such as the monocyte chemoattractant protein-1 (MCP-1), IL-6, and IL-8, exacerbates the infiltration of macrophages into the adipose tissue and secretion of IL-1β and TNFα. This, in turn, lowers the expression of insulin receptor substrate 1 (IRS-1) and glucose transporter type 4 (GLUT4) and leads to the development of insulin resistance [14,15]. Moreover, PPARγ and CCAAT/enhancer-binding protein alpha (C/EBPα) represent the most critical players in maintaining adipocyte homeostasis, and their expression was found to be altered when impaired adipogenesis occurs [16]. Therefore, identifying molecular mediators of insulin resistance in non-obese individuals may aid in reversing insulin resistance before the onset of T2D. Among the potential molecular targets underlying adipogenesis impairment is the high expression of the anti-adipogenic transcription factor GATA Binding Protein 3 (GATA3) [17]. 

Previous studies have shown that GATA3 suppresses the transition from preadipocytes to adipocytes by inhibiting the expression and activity of PPARγ2 and C/EBPs [18,19,20,21,22,23]; however, most of these studies have focused on the benefit of adipogenesis inhibition in reducing obesity. Other studies have suggested the GATA3′s crucial function as a gatekeeper of terminal adipocyte differentiation [24], and that its inhibition may reverse the impaired adipogenesis and linked insulin resistance. Therefore, in a previous study, we investigated the inhibition of GATA3 using a new class of antisense molecules known as DNAzymes [25] to rescue adipogenesis and improve insulin signaling. Pioglitazone was used as a positive control in our study. De Souza et al. reported the effect of pioglitazone on adipose tissue’s physiology, accumulation, and distribution in female Zucker rats. Insulin resistance and hyperlipidemic states decreased with pioglitazone, whereas food consumption and whole-body adiposity increased. The study revealed that the increase in adiposity occurred throughout the body. Analyzing adipocyte sizing profiles, DNA content, and fat histology indicated an increase in the number of new small adipocytes and a shrinkage or/and disappearance of existing mature adipocytes [26]. Our results indicated that such inhibition indeed improved adipocytes differentiation, modulated the cytokine profile, and improved insulin sensitivity in insulin resistant cells [27]. In this study, we investigated the role of targeting GATA3 expression in vitro and in vivo on modulating adipogenesis, oxidative stress, inflammation, and insulin signaling. This is the first proof-of-concept study aimed at showing that the inhibition of GATA-3 expression can induce adipogenesis in human primary preadipocytes and at the site of treatment in non-obese normal mice, testing the hypothesis that GATA3 inhibition can lead to healthier fat redistribution. 

## 2. Results

### 2.1. Effect of GATA3 Inhibition on Preadipocyte Proliferation, Adipogenic Capacity, Gene Expression, and Insulin Signaling 

**Figure 1:**
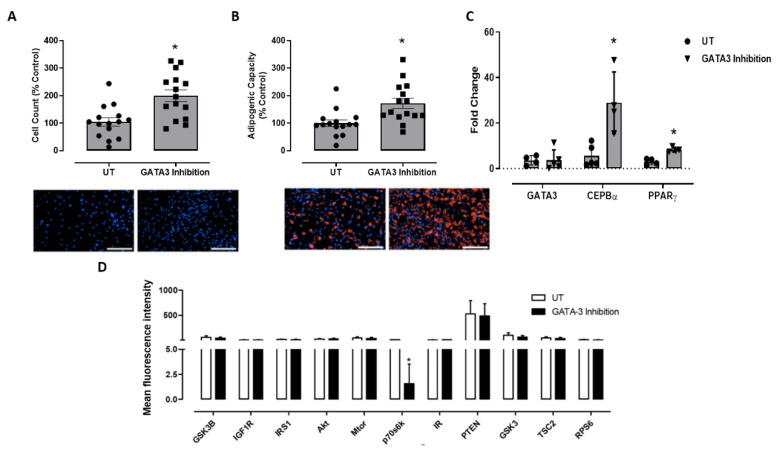
Effect of GATA3 inhibition on primary adipocytes (**A**) proliferation, (**B**) adipogenic capacity, (**C**) gene expression, and (**D**) insulin signaling. Data are presented as mean ± SEM with representative images below each bar (**A**,**B**). The tests were conducted on 4 independent biological replicates, with at least 3 technical replicates of each. Statistical analysis for effect of GATA3 inhibition was determined by Mann–Whitney U test (* *p* < 0.05) for (**A**–**C**) and two-way ANOVA followed by Bonferroni Posttests (* *p* < 0.05) for (**D**). Scale bars represent 200 μm.

We previously showed that GATA-3 inhibition causes reduction in GATA-3 expression in 3T3L-1 mouse preadipocytes after 48 h of treatment [27]. GATA3 inhibition was validated in primary human preadipocytes isolated from five BFP biopsies. The results showed that treating preadipocytes with GATA3 inhibitor caused an increase in the cell number (Figure 1A) and adipogenic capacity with more mature adipocytes (1.8 Fold increase) in the GATA3 inhibitor-treated group compared to the untreated group (Figure 1B). Moreover, a significant increase in the expression levels of the adipogenic genes (PPARγ, CEPBβ) from the preadipocytes treated with the GATA3 inhibitor was observed (Figure 1C). In order to assess the effect of GATA3 inhibition on insulin signaling, we also measured the phosphorylation levels of different insulin response-associated kinases. The results showed a significant reduction in p70S6K phosphorylation level from the GATA3 inhibitor-treated group compared to the untreated group (Figure 1D).

### 2.2. Effect of GATA3 Inhibition on Total Animal and Tissue Weight

**Figure 2:**
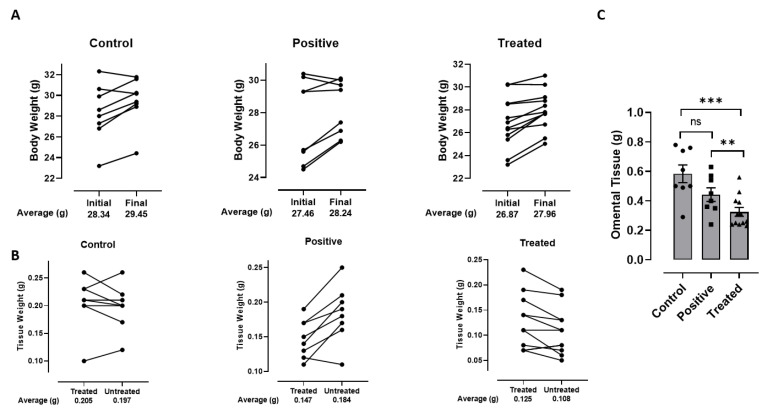
In vivo effect of GATA3 inhibition on (**A**) total animal weight between start and end of experiment, (**B**) weight of SAT tissue between treated (site of injection) and untreated (control) sites, and (**C**) weight of omental tissue. Bar graphs show paired data for each mouse for n = 6–12 animals per group. Statistical analysis was determined by paired (**A**,**B**) and unpaired (**C**) *t*-test (** *p* < 0.02, *** *p* < 0.01).

Twenty-eight animals were divided among three groups: the Vehicle Control (N = 8), Positive Control (N = 8), and GATA3 inhibitor-treated (N = 12) Group. Repeated treatment of animals with 10 µg/0.1 mL of DNAzyme over a period of 2 weeks showed a significant increase in animal total weight at the end of the study compared to the animal weight at the beginning of the study (Figure 2A) in all groups. Control group showed a 1.04-fold (*p*-value 0.020246) increase in animal weight, with positive and treated groups showing a 1.03-fold (*p*-value 0.049796), and 1.04-fold (*p*-value 0.000469) increase, respectively. When comparing the weight difference between the right (treatment site) and left (opposite control side) SAT, there was a significant increase in the right adipose tissue weight from the GATA3 inhibitor-treated group of 1.3-fold (*p*-value 0.02), but not in the control group (*p*-value 0.4). However, the positive control group showed a significant decrease in the weight of the right adipose tissue of 0.9-fold (*p*-value 0.004) (Figure 2B). As reported by de Souza et al., the remodeling effect of pioglitazone on adipose tissue is time dependent. The existing mature adipocytes will start to shrink or/and disappear, followed by the appearance of new adipocytes as indicated below (Figure 2B) [26]. Furthermore, there was a statistically significant reduction in the omental adipose tissue weight between the GATA3 inhibitor-treated group and the vehicle control groups of 0.6-fold (*p*-value 0.003), and between the GATA3 inhibitor-treated and positive control groups of 0.7-fold (*p*-value 0.02) (Figure 2C). However, there was no statistically significant difference in the omental weight between the vehicle control and positive control groups (Figure 2C). 

### 2.3. Effect of GATA3 Inhibition on SOD and Catalase Levels in Animal Sera

**Figure 3:**
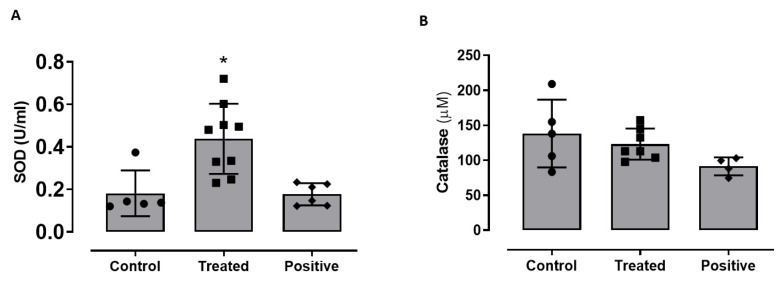
(**A**) SOD and (**B**) catalase levels detected from mice serum. Data are presented as mean ± SEM for n = 4–12 animals. Statistical analysis for was determined by One-way ANOVA (* *p* < 0.05).

There was a significant increase in SOD activity in the GATA3 inhibitor-treated group compared to the vehicle control group, but no significant difference between the positive control and vehicle control groups (Figure 3A). Moreover, there was no significant difference in the catalase levels among the three groups (Figure 3B).

### 2.4. Effect of GATA3 Inhibition on Gene Expression Levels from Different Adipose Tissues (Right, Left, and Omental Sites)

**Figure 4:**
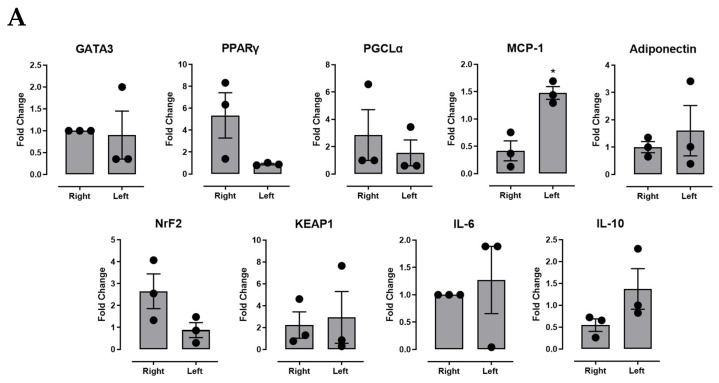

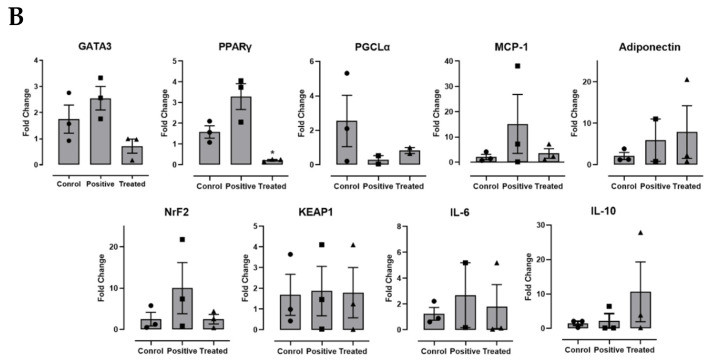
Gene expression in the stromal vascular fraction derived from GATA-3 inhibitor-treated animals. (**A**) SAT at right (treatment site) and left (opposite control side) sites and (**B**) omental adipose tissue in vehicle control, positive control, and GATA-3 inhibitor-treated animals. Differences in gene expression levels were compared for GATA3, PPARγ, PGC1alpha, MCP-1, Adiponectin, NrF2, KEAP-1, IL-6, and IL-10. Data are presented as mean ± SEM for n = 6 replicates from 3 animals each. Statistical analysis for was determined by (**A**) paired *t*-test and (**B**) ANOVA (* *p* < 0.05).

In terms of gene expression from the SVF derived from different adipose tissue sites (SAT and omental), we found that GATA3 inhibition decreased the expression levels of MCP-1 in the right (injection site) compared to opposite SAT site (Figure 4A). In addition, the inhibition of GATA3 caused a reduction of PPARγ expression levels in the omental site compared to the vehicle control group (Figure 4B). No difference in gene expression was seen between the right (injection site) and left (opposite control side) tissues in the vehicle and positive control groups. 

## 3. Discussion

Various studies have suggested that non-obese individuals could equally become insulin resistant and develop T2D if left untreated, with the term metabolically obese apparently healthy individuals being used to describe insulin-resistant non-obese individuals [3,8,28]. Modulators, including GATA3, were shown to be highly expressed in insulin-resistant tissues and to be responsible for preventing adipogenesis. Despite its potential role in obesity prevention, such approach has a great risk of preventing adipogenesis, which is required to maintain adipose tissue homeostasis and insulin sensitivity [29]. GATA3-associated impaired adipogenesis affects lipid homeostasis contributing to body fat distribution, causing the deposition of ectopic fat in the liver, kidney, and skeletal muscles; triggering insulin resistance; and increasing the risk of T2D [29]. Conversely, our recent studies have shown that targeting GATA3 expression could provide an alternative strategy for inducing adipogenesis at healthy fat depots, in addition to the modulation of inflammation and oxidative stress [27,30]. The emerging data provide a confirmation of the pro-adipogenic and insulin-sanitizing effect of GATA3 inhibition in primary human adipocytes derived from non-obese individuals. Moreover, our data provide a further proof of concept showing that inhibition of GATA-3 expression in non-obese animals can induce adipogenesis at site of treatment and test the hypothesis whether it can lead to fat redistribution. Pioglitazone was used as a positive control shown previously to trigger a decrease in the ratio of visceral to subcutaneous fat [31]. 

Recent reports have suggested that both preadipocytes and mature adipocytes play an equally important role in the maintenance of adipose tissue hemostasis and the development of insulin resistance when dysfunctional [32]. These reports were consistent with our findings showing an increased cell number and adipogenic capacity in the GATA3 inhibition group, indicating the presence of active adipogenesis and differentiation of preadipocyte to mature adipocytes. To elucidate the roles of adipogenesis in the early development of insulin resistance, we characterized the gene expression profile in human primary adipocytes. Previous studies have reported that down-regulation of PPARγ/CEPBβ was observed in preadipocytes isolated from insulin resistant individuals [33]. Other studies have shown that adipocyte differentiation was compromised under these conditions; thus, using modulators such as GATA3 inhibitor could improve adipogenesis and correct insulin signaling in adipose tissue from insulin-resistant individuals [34]. Our results indicated that PPARγ and CEPBβ genes were differentially expressed in these cells, with high expression levels seen in the GATA3 inhibition group compared to the cells in the untreated group. 

Impaired adipogenesis could affect the levels of different proteins and kinases involved in insulin signaling pathway [3]. Our in vitro results showed a reduction in the phosphorylation of the p70S6K from primary adipocytes treated with the GATA3 inhibitor. However, our in vivo p70S6K (ribosomal protein S6 kinase 1, p70S6K,) is a serine kinase that was reported to inhibit the function of IRS-1 by facilitating its degradation, thus inhibiting insulin signaling [35]. Previous in vivo studies showed that knocking out p70S6K in mice protected them from diet-induced insulin resistance. Moreover, obese mice were shown to have elevated p70S6K activity in the adipose tissue, skeletal muscles, and liver, which are strong indicators that could contribute to insulin resistance [35]. Together, these findings highlight the important role GATA3 plays in the development of impaired adipogenesis and insulin resistance, therefore blocking GATA3 could reverse these mechanisms and enhance both adipogenesis and insulin signaling. 

In order to validate the effect of GATA3 inhibition in vivo, we carried out a proof-of-concept study utilizing normal-weight mice. Our findings demonstrate that there was an increase in the total animal weight at the end of the experiment from the GATA3 inhibitor-treated group. The increase in the animal weight was accompanied by an increase in the weight of the tissues dissected from the right site (injection site) compared to the left-site tissues. Inhibiting GATA3 in the injection sites might have enhanced the mobilization, recruitment, and differentiation of adipocyte progenitor cells to the right site, therefore promoting adipogenesis and causing an increase in the tissue weight. In contrast, the GATA3 inhibitor-treated group showed a reduction in the omental adipose tissue weight compared to the other groups, perhaps accounting for the increased weight of fat tissue at the injection site in treated animals. Such reduction was marked by a decrease in the expression levels of PPARγ. These data suggest a protective effect associated with GATA3 inhibition, which is in agreement with previous studies associating the omental adipose tissue mass/size with the amplified inflammatory status and insulin resistance [36]. Moreover, other studies correlated omental adipose tissue size with the degree of insulin resistance [13]. A similar finding was found in the pioglitazone-treated animals where the visceral fat area and ratio of omental to SAT fat decreased. Interestingly, there was a decrease in SAT fat mass in the pioglitazone-treated animals. This unexpected result could be due to the local administration of pioglitazone, whereas previous studies have shown a systemic administration causing increased subcutaneous but reduced visceral adipogenesis [31]. This interesting observation requires further investigation to explain the discrepancy in the effect of pioglitazone.

Impaired adipogenesis is characterized by having an imbalance between the oxidative and antioxidative markers. SOD, a major antioxidant enzyme that protects adipocytes during proliferation and differentiation, was found to be elevated in tissues and cells undergoing active adipogenesis and cellular differentiation [37]. Impaired adipogenesis causes a reduction in the SOD levels, increasing the oxidative stress in the adipose tissues. Therefore, having an antioxidant modulator might restore the balance between the oxidative and antioxidative markers. Our results showed that GATA3 inhibition caused an increase in the SOD levels measured from the animals’ serum, which correlates with the increase in the animals’ total weight indicating the presence of active adipogenesis [37].

The proinflammatory profile of dysfunctional adipose tissue plays a critical role in lowering the adipogenic capacity of preadipocytes, leading to a reduction of the lipid storage space and elevation in ectopic lipid accumulation [38]. To validate this point, we measured to what extent GATA3 inhibition could account for the expression of MCP-1 [38]. MCP-1 is the most extensively studied CC chemokine linked to etiologies of obesity-related insulin resistance and T2D [39]. Studies have shown that MCP-1 is overexpressed in obese and insulin-resistant animals, suggesting that the elevation in the MCP-1 levels could reduce adipocyte differentiation, alternatively causing metabolic abnormalities associated with obesity, as well as hyperinsulinemia (e.g., T2D) [40]. In addition, MCP-1 is a well-known potent inflammatory chemokine that recruits macrophages; thus, targeting it could prevent macrophage-induced inflammation in adipose tissue [39]. Our results showed that expression levels of MCP-1 at the site of GATA3 inhibitor injection were significantly lower than the opposite side. This suggests that GATA3 inhibition has the potential to reduce macrophage infiltration associated with adipose tissue inflammation seen in impaired adipogenesis.

However, this study has potential limitations, including the need to verify whether the increased adipose tissue weight at the injection site is due to increased fat content or adipocyte number. In addition, since this in vivo study is a proof of concept, the used animal models were healthy mice with normal insulin signaling. Hence, the effect of GATA3 inhibition on activation (phosphorylation) of insulin signaling was not investigated. These limitations must be addressed in future studies using diseased models with insulin resistance to explore the effect of GATA3 inhibition effect on reversing IR in vivo. 

In conclusion, our in vitro and in vivo data indicate that inhibiting GATA-3 expression restores adipogenesis and fat distribution, improves adipocytes differentiation, and enhances insulin signaling. Our data therefore suggest the potential utilization of GATA-3 modulation for preventing the development of insulin resistance in non-obese as well as obese individuals. However, despite holding great potential and being a promising modulator, such approach requires further investigation and validation in relevant animal disease models.

## 4. Materials and Methods

### 4.1. In Vitro Effect of the GATA3 Inhibition

Recruitment criteria of participants were previously described [17]. Approvals of the Institutional Research Board (IRB) committees of Hamad Medical Corporation and Qatar University for the proposed project were sought before the onset of research (MRC-03-21-154, and QU-IRB 1548-EA/21). Five patients undergoing maxillofacial surgeries were recruited, and information about the donors’ gender and BMI were collected. Stromal vascular fractions (SVFs) were isolated from buccal fat pad (BFP) biopsies collected from the recruited subjects as described. SVF were re-suspended in stromal media containing DMEM-F12 with 10% FBS, 1% Antibiotic-Antimycotic solution and 1% L-Glutamine (200 mM) and plated at 4 × 10^4^/cm^2^. The cells were then maintained in a humidified incubator at 37 °C with 5% CO_2_. The media was changed every 2–3 days until the cells achieve 80–90% confluence. When confluent, cells were either harvested or induced to differentiate by changing the medium into differentiation medium (DMEM-F12, 3% FBS, 1% Antibiotic-Antimycotic solution, 1% L-Glutamine (200 mM), 1 µM dexamethasone, 0.25 mM IBMX, 0.2 µM Insulin, Biotin (66 µM), Rosiglitazone (PPARγ agonist) (5 µM))) for 3–7 days, followed by 9–10 days in maintenance medium containing the same components as the differentiation medium, excluding IBMX and rosiglitazone as described previously [41]. To investigate the effect of GATA3 inhibition, cells were grown as described above and treated with GATA3 inhibitor as mentioned previously [27]. Briefly, 24 h after seeding, the cells were transfected with 1 ng/µL hgd21 (human GATA3 mRNA specific DNAzyme) and Lipofectamine 3000 transfection reagent. The cells were incubated for 6–8 h, and the media was then changed to induce adipogenic differentiation. 

#### Assessment of Cell Viability and Adipogenic Capacity

Cells were fixed with 4% formaldehyde (Thermo Scientific, Waltham, MA, USA, 28908) and stained with DAPI (Molecular probes by life technologies, D1306) and Lipidtox (Invitrogen, H34476) as previously described [27]. Total number of nuclei (DAPI-positive) and differentiated adipocytes (Lipidtox-positive) were automatically scored in 25 fields/well by Cytation 5 Cell Imaging Multi-Mode Reader (Agilent Technologies, Santa Clara, CA, USA). Adipogenic capacity was assessed by calculating the percentage of Lipidtox-positive cells to the total number of nuclei. 

### 4.2. In Vivo Assessment of the GATA3 Inhibition

#### 4.2.1. Liposomes Preparation

Liposomes were prepared using the ethanol-based proliposome technology by adapting a previously published protocol [42]. Briefly, 50 mg of phospholipid was mixed with 100 µL of absolute ethanol and dissolved at 70 °C. Then, 50 mg of cholesterol was added to the previous mixture and dissolved at 70 °C water bath. After, 1 mL of 0.1 mg/mL DNAzyme hgd40 (mouse GATA3 mRNA specific DNAzyme) was added to the phospholipid-cholesterol mixture with continuous vigorous mixing for 4 min. The resulting blend was left at room temperature for 2 h, followed by sonication for 10 min. The liposomes were centrifuged at 12,000 rpm for 15 min to eliminate the titanium particles released by the probe of the sonicator. 

#### 4.2.2. Assessment of Insulin Signaling

Insulin signaling was measured by assessing the phosphorylation levels of IRS-1, GSK3B, IGF1R, Akt, Mtor, p70s6k, IR, PTEN, GSK3, TSC2, and RPS6 in total cell lysates (equal volume) as previously described (Al-Mansoori, Al-Jaber et al. 2020), using a commercial Bio-Plex Pro™ Cell Signaling Akt Panel (Bio-Rad, Heracles, CA, USA) using Luminex 200 technology (Thermo Fisher Scientific, Waltham, MA, USA) following the manufacturer’s instructions. The Bio-Plex Pro cell signaling assays sensitivity, as well as the intra- and inter-assay %coefficient variation per species, can be found at https://www.bio-rad.com/webroot/web/pdf/lsr/literature/Bulletin_6285.pdf (accessed on the 17 September 2022).

#### 4.2.3. Animal Care, Experimental Design and Treatment

Adult normal weight male (12–16 weeks old) BALB/c mice were provided by the Laboratory Animal Research Center (LARC) at Qatar University (QU). Animals were housed in individually ventilated cages (IVC) under standard husbandry conditions (room temperature 18–22 °C, relative humidity 40–65% and 12/12 h light/dark cycle), provided with normal chow diet and drinking water ad libitum. All animal procedures were performed according to approved institutional ethical rules and regulations and were approved by Qatar University-Institutional Animal Care and Use Committee (QU-IACUC 024/2020). A total of 28 animals was used in this study and divided into three groups: (A) Vehicle Control Group, with 8 animals that were treated with 100 µL of DNAzyme-free liposomes; (B) Positive Control Group, with 8 animals that were treated with liposome-loaded with 1 µM of pioglitazone (40 mg/Kg); and (C) GATA3 inhibitor-treated Group, with 12 animals treated with liposome-loaded DNAzyme (10; µg/mL, hgd40). Treatments were administered subcutaneously to the right flank region (site of injection), twice a week for 2 weeks. The mice were housed under standard animal husbandry conditions with 12 h dark and light cycle and were provided standard rodent chow and water ad libitum. Animals were weighed at the beginning and the end of the study. All the animals were euthanized as per AVMA guidelines, and the subcutaneous adipose tissues from right flank (site of injection), left flank (opposite site), and omental were collected from scarified mice, weighed. Blood was drawn via a cardiac puncture. 

#### 4.2.4. Assessment of Oxidative Stress

Oxidative stress was assessed by measuring the activity of antioxidant enzymes, including catalase and superoxide dismutase (SOD), in serum samples prepared from collected blood using the Catalase Assay Kit (Merck Millipore), and the SOD kit (Merck Millipore, Singapore) following the manufacturer’s instructions. Measurements and data analysis was performed using the Cytation 5 Cell Imaging Multi-Mode Reader (Agilent Technologies, Santa Clara, CA, USA).

#### 4.2.5. Assessment of Gene Expression from Both in Vitro and in Vivo Experiments

For the in vivo experiments, SVFs were isolated from adipose tissue biopsies collected from right/left thighs and omental depots using established protocol [43,44,45]. Briefly, the collected adipose tissue biopsies (0.5 g) were homogenized using gentleMACS™ Dissociator (Miltenyi Biotec, Bergisch Gladbach, Germany). Then, the biopsies were digested using collagenase solution (0.1% collagenase I/1% BSA in PBS) for 1 h at 37 °C. Samples were then centrifuged at 1500 rpm for 5 min to separate SVF. The resulting cell pellet was then washed with 1% BSA, followed by erythrocyte lysis buffer for 10 min. TRizol reagent (Invitrogen) was added to the pellet for the RNA extraction using the TRizol method according to the manufacturer’s instructions. For the in vitro experiments, RNA was extracted from preadipocyte cultures before and after the induction of differentiation using TRIzol method (Invitrogen) according to the manufacturer’s instructions. Then, 3 μg of the resulting RNA from in vitro and in vivo experiments was used for first-strand cDNA synthesis using the Superscript III first-strand synthesis super mix kit (Invitrogen, Waltham, MA, USA) according to the manufacturer’s instructions. Real-time PCR was carried out for gene expression analysis using 10 ng of the produced cDNA with the listed primers (Table S1) using the 7500 Real-Time PCR System from Applied Biosystem. The PCR conditions were as follows: 1 cycle of 95 °C for 10 min, 45 cycles of 95 °C for 15 s, 55 °C for 40 s, 72 °C for 30 cycles, and finally, 60 °C for 15 s. Real-time PCR was carried out in triplicate, and the GAPDH was used as a housekeeping gene for normalization of the amplified signals of the target genes. The data analysis was performed using the ΔΔCt-based calculations [46]. 

### 4.3. Statistical Analysis

Comparisons were performed using a *t*-test, one-way ANOVA, two-way ANOVA, or linear.

## 5. Patents

The authors declare a patent involved in the reported work (US Patent App. 16/909,755).

## Data Availability

The datasets used and/or analyzed during the current study are available from the corresponding author on reasonable request.

## Supplementary Materials

The supporting information can be downloaded at: https://www.mdpi.com/article/10.3390/ijms231911142/s1.
