# Supplementary material for: In Vitro and In Vivo Validation of GATA-3 Suppression for Induction of Adipogenesis and Improving Insulin Sensitivity
Source: Int J Mol Sci. 2022 Sep 22;23(19):11142. doi: 10.3390/ijms231911142 (PMC9569927; doi:10.3390/ijms231911142)
Supplement: Supplementary file 1 [file ijms-23-11142-s001.zip › ijms-1874664-supplementary.pdf]

Supplementary Materials

Table S1. the list of forward and reverse primers used for gene expression analysis

Gene	Primers Sequences (5′ to 3′)
**GAPDH**	*f:* AGGTCGGTGTGAACGGATTTG
	r: TGTAGACCATGTAGTTGAGGTCA
**GATA-3**	*f:* GAACCGGCCCCTTATCAAG
	r: ACAGTTCGCGCAGGATGTC
**PPAR-γ**	*f:* GGCTTCCACTATGGAGTTCA
	r: GATCCGGCAGTTAAGATCAC
**PGC1-α**	*f:* TGCAGCCAAGACTCTGTATG
	r: ATTGGTCGCTACACCACTTC
**MCP-1**	*f:* GCTACAAGAGGATCACCAGCAG
	r: GTCTGGACCCATTCCTTCTTGG
**ADIPONECTIN**	*f: ACTGCAACTACCCATAGCCCAT*
	r: TGTCGACTGTTCCATGATTCTCC
**NrF2**	*f:* CTGAACTCCTGGACGGGACTA
	r: CGGTGGGTCTCCGTAAATGG
**KEAP-1**	*f:* CAGCAGTTAAGGGCACCAATGC
	r: CCTGCCTCAGCTTCCCATCA
**IL-6**	*f:* TAGTCCTTCCTACCCCAATTTCC
	r: TTGGTCCTTAGCCACTCCTTC
**IL-10**	*f:* GCTCTTACTGACTGGCATGAG
	r: CGCAGCTCTAGGAGCATGTG
